# Health System Strengthening Through Professional Midwives in Bangladesh: Best Practices, Challenges, and Successes

**DOI:** 10.9745/GHSP-D-23-00081

**Published:** 2023-10-30

**Authors:** Farida Begum, Rowsan Ara, Amirul Islam, Stephanie Marriott, Anna Williams, Rondi Anderson

**Affiliations:** a United Nations Population Fund, Dhaka, Bangladesh.; b Liverpool School of Tropical Medicine, Liverpool, England.; c Data, Design + Writing, Oregon City, Oregon, USA.; d United Nations Population Fund, Aleppo, Syria.

## Abstract

In 2008, a cadre of professional midwives was introduced in Bangladesh. Since then, 120 midwifery educational programs have been established. There are 2,556 midwives serving at 667 government health facilities, and there are more midwives working in nongovernmental organizations and the private sector. This case study documents the process of establishing a midwifery profession with distinct midwifery expertise in Bangladesh and aims to guide other low- and middle-income countries in best practices and challenges. We describe the national administrative groundwork for the profession's launch, roll-out of an education program aligned with the International Confederation of Midwives, national deployment, enabling environments in deployment, and the professional association. Bangladesh's professional midwives' roles in humanitarian response and the COVID-19 pandemic are also discussed. The first and final authors were closely involved in supporting the government's establishment of the profession, and their direct experience is drawn upon to contextualize the topics. In addition, the authors conducted a desk review of documents that supported the profession's integration into the health system and documented its results. Both routine program data and existing research studies were reviewed. Outcomes show that midwives are deployed to 95% of government subdistrict hospitals. About 50% of these hospitals are fully staffed with 4 midwives, and within the hospitals, midwives are in charge of 90% of the maternity wards and attend 75%–85% of the births. Since the midwives' deployment, significant quality improvement for most World Health Organization indicators has been found, along with increases in service utilization. The experience of establishing a new midwifery profession in Bangladesh shows that it is possible for a lower middle-income country to introduce a globally standard midwifery profession, distinct from nursing, to improve quality sexual, reproductive, maternal, newborn, and adolescent health services in both humanitarian and development settings.

## Abstract (precis)

The authors detail the establishment of the profession of globally standard midwives deployed into the national health care system in Bangladesh that improved the quality and availability of sexual, reproductive, maternal, newborn, and adolescent health services.

## Key Messages

- Implementation of a cadre of professional midwives into Bangladesh's health care system involved designating midwifery posts, developing a regulatory system and globally standard midwifery education, and creating enabling workplace settings.
- Midwifery leadership is still nascent, and ongoing support for enabling environments for midwifery is necessary for quality, lifesaving midwifery-led care.
- Nationally deployed midwives are a cost-effective, sustainable quality improvement intervention for women's health in Bangladesh.
- Lessons learned and best practices from introducing professional midwives in Bangladesh may provide helpful insight for other countries.

## BACKGROUND

### Ensuring Quality Through Professional Midwifery

Gaps in access to comprehensive sexual, reproductive, maternal, newborn, and adolescent health (SRMNAH) services among the world's poorest are ubiquitous.^1^^,^^2^ Skilled birth attendants and facility births have not adequately improved outcomes. Ensuring quality services is now at the forefront of the response.^2^ The International Confederation of Midwives (ICM) has set global SRMNAH competencies for professional midwives.^3^^–^^6^ Analysis shows that midwives—if educated, regulated, enabled, and integrated within an interdisciplinary team—could meet about 90% of the need for SRMNAH services.^7^ By 2030, 750,000 new midwives are required to meet projected SRMNAH needs.^8^ Thus, further investment in midwives is called for to achieve the Sustainable Development Goals.^3^ Indeed, it is estimated that an increase in enabled midwives could avert 41% of maternal deaths, 39% of neonatal deaths, and 26% of stillbirths.^9^

Many countries have not established midwifery education that is in accordance with ICM standards and that meets ICM competencies for their basic SRMNAH provider. Low-income countries often use less-educated cadres for this role.^8^ In addition, maternity providers often operate in generalist posts, reducing their time dedicated to SRMNAH.^8^^,^^10^ Regulatory bodies, education programs, and post designations frequently are not specific to midwives, and when they are, resistance to midwifery care from doctors and nurses is common.^8^^,^^11^ As quality midwifery practices are often new to health systems, specialization and leadership of confident experts are needed to make sustainable change.

### Midwifery in Bangladesh

This case study details the introduction of an ICM-aligned midwifery cadre between 2008 and 2022 in Bangladesh, building upon the history described by Bogren et al.^12^ Bangladesh has 47 million women of reproductive age. It is a lower middle-income country that has shown marked improvements in maternal mortality but is not on target to meet Sustainable Development Goals 3 and 5. The health system is complex, with actors from the public sector, private sector, nongovernmental organizations (NGOs), and United Nations agencies. Three-quarters of the poorest women give birth at home. For affluent women, care with a private doctor and cesarean deliveries are the norm and are rapidly rising, leaving gaps in many options for quality maternity care.^12^^–^^14^

Historically, midwifery in Bangladesh was part of both nursing and primary care.^12^ Public maternity wards were previously staffed with generalist nurse-midwives and community health workers, neither of whose education aligned with ICM competencies.^15^ Program assessments found gaps in their basic competencies.^12^

In 2008, Bangladesh took the initiative to upgrade its midwifery workforce to meet global standards. When midwives were initially deployed, they faced significant resistance from hospital managers, doctors, and nurses to their leading maternity care provision due to lack of understanding of the midwifery scope of practice in the public sector.^10^^,^^16^ Over time, intensive investment in on-site mentoring in public facilities and the profession's increased national profile contributed to greater acceptance of midwifery care among both providers and the communities midwives serve. Currently, there are 8,000 registered midwives, of which 2,556 are deployed in 667 government hospitals and health centers in distinct midwife posts. More than 300 midwives are deployed through NGOs in humanitarian settings, and many of the remaining work in private facilities. In the private sector, midwives often work as nurses because midwifery posts are not designated.^16^ In 2023, most midwives deployed in public facilities are in charge of the labor ward and attend 75%–85% of births.^17^ Published research shows statistically significant increases in evidence-based birth practices (e.g., use of antenatal card and partograph, upright birth positioning, and skin-to-skin contact between mother and newborn) in public facilities that have midwives, indicating that midwives play a role in care quality improvement.^18^^,^^19^ Based on World Health Organization (WHO) estimates for the needed human resources,^20^ the Ministry of Health and Family Welfare (MOHFW) has approved 5,000 more midwifery posts, and an additional 20,000 are awaiting approval.

In Bangladesh, investment in on-site mentoring in public facilities and the midwifery profession's increased national profile contributed to greater acceptance of midwifery care among providers and the communities midwives serve.

This case study documents the process of establishing a midwifery profession with distinct expertise to help guide other low- and middle-income countries in best practices and challenges. It draws on the experience of the lead author, who supported and worked within the government to establish the midwifery profession as either a government, United Nations Population Fund (UNFPA), or WHO employee since the inception of the midwifery profession, as well as the final author's experience since her deployment with UNFPA in 2015. Starting in August 2022, the authors conducted a desk review, analyzed routine program data and research study results, and held a series of conversations to share and validate the historical events. The authors' direct experiences complemented the desk review and informed the structure of the narrative, as well as the presentation of successes and challenges.

## ESTABLISHING THE MIDWIFERY PROFESSION IN BANGLADESH

**FIGURE 1:**
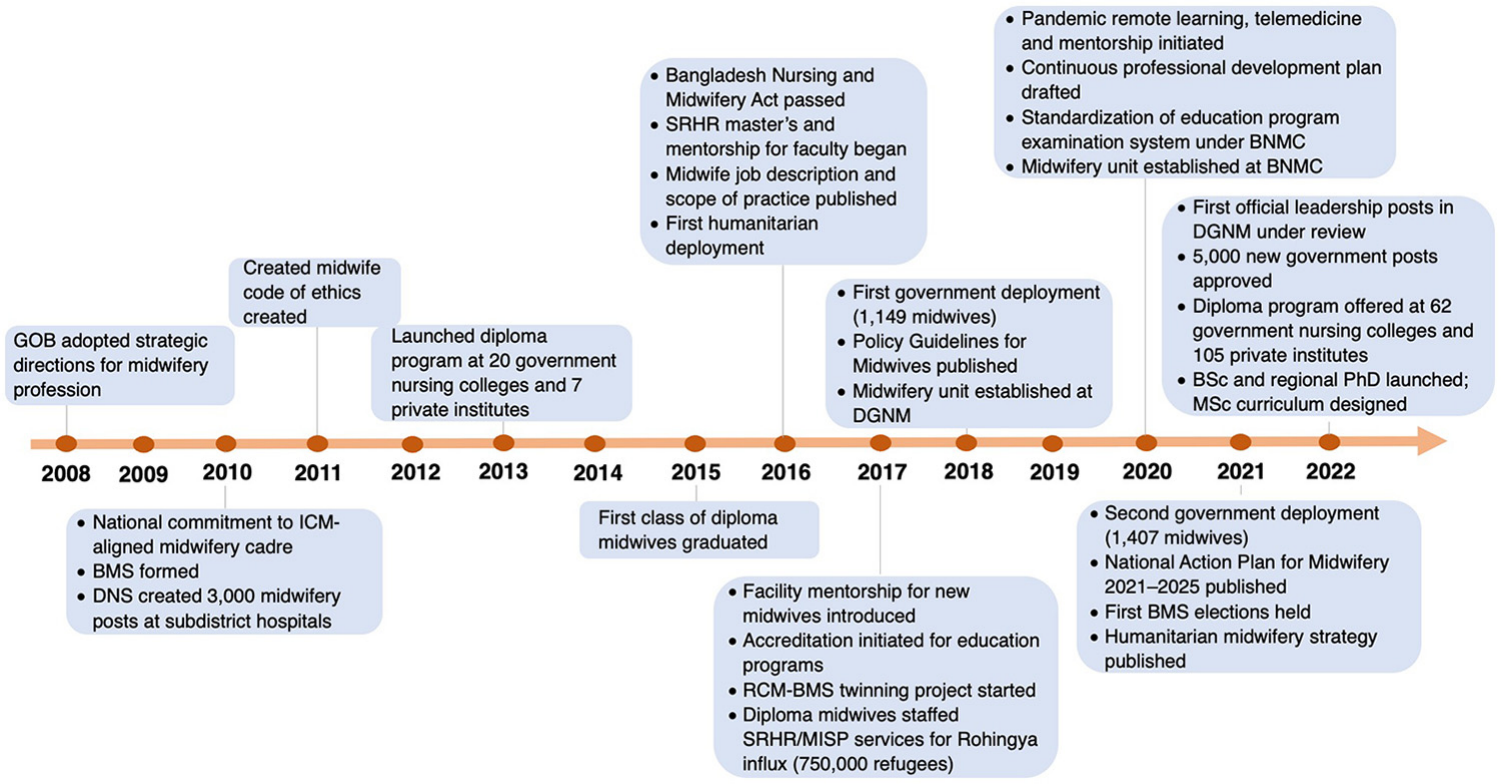
Timeline of Development of Midwifery Profession in Bangladesh Abbreviations: BMS, Bangladesh Midwifery Society; BNMC, Bangladesh Nursing and Midwifery Council; BSc, Bachelor of Science; DGNM, Directorate General of Nursing and Midwifery; DNS, Directorate of Nursing Services; GOB, Government of Bangladesh; ICM, International Confederation of Midwives; MISP, Minimum Initial Service Package; MSc, Master of Science; PhD, Doctor of Philosophy; RCM, Royal College of Midwives; SRHR, sexual and reproductive health and rights.

The structure of this case study was informed by both the UNFPA and ICM midwifery frameworks,^21^^,^^22^ which were combined as follows to best reflect the Bangladesh experience: national administrative groundwork, education, association, workforce, and enabling environment. In the current ICM professional framework, gender equality cuts across all core program pillars. Issues around gender equality were similarly addressed following an integrated approach within each program area. The timeline (Figure 1) provides a high-level overview of milestones achieved in the profession's establishment.

### National Administrative Groundwork

Ensuring that enabling administration and regulation are in place is essential groundwork for a strong midwifery profession. Before education or deployment can be realized, administration and regulation must establish and define the midwifery profession.^6^

#### Governance

After the Prime Minister's commitment of 3,000 midwives by 2015 given at the 2010 “Every Woman Every Child Initiative” during the United Nations General Assembly, multistakeholder discussions moved the vision of globally standard midwives forward. The MOHFW took broad governance steps to give midwives a legal domain and a service grade within the health system. With support from nursing, the MOHFW created 3,000 distinct midwife posts and determined where midwives would be educated and subsequently deployed. Although advocacy efforts have been made, currently, there is no systemwide creation of midwifery posts in the private sector. In the public sector, there are 2 related directorates. The health services directorate oversees the current midwives' workplace environment, including the hospital managers and interdisciplinary teams. The family planning directorate has approved midwife posts, but their deployment is awaiting the needed administrative process.^12^^,^^23^

Implementation details, including policies and guidelines, originated from the Directorate of Nursing Services, which, in 2016, became the Directorate General of Nursing and Midwifery (DGNM) and deploys midwives within the government health system. The nursing regulatory body became the Bangladesh Nursing and Midwifery Council (BNMC), authorizing both public and private midwifery education and licensing. BNMC introduced the Bangladesh Nursing and Midwifery Act, curricula, licensing, accreditation, monitoring, and a path for continuing education linked to licensing.

The BNMC introduced the Bangladesh Nursing and Midwifery Act, curricula, licensing, accreditation, monitoring, and a path for continuing education linked to licensing.

Initially, United Nations agencies (including WHO and UNFPA), their respective donors (notably the Swedish International Development Cooperation Agency; the United Kingdom Foreign, Commonwealth & Development Office; and Global Affairs Canada), and NGO and academic partners provided technical support, advocacy, and funds. UNFPA supported 2 international midwifery specialists to work with the government to ensure a globally aligned vision.^12^

In 2010, the Bangladesh Midwifery Society (BMS) (see section on Association) was formed with nursing faculty to represent the new cadre and played a crucial role in championing the new cadre. The Obstetric and Gynecological Society of Bangladesh supported BMS and ensured that the country viewed midwives as an important addition to the health system.^12^

Currently, formalized leadership roles for midwives are nascent. With a maximum of 6 years of experience, professional midwives have only recently entered the prerequisite higher education needed for leadership roles. A midwifery career path and organogram have been submitted to the MOHFW. Four midwives have recently been deployed to the DGNM to lead the profession.^16^

#### Policy Formulation

Development of the regulatory system consisted of establishing a code of ethics and standard operating procedures, as well as licensing, registration, and practice guidelines, and the needed administration for education programs. Though developed through thoughtful multistakeholder deliberation, many guidelines needed revision over time as understanding of the professional midwife role and the needed government guidance evolved.^12^

For example, the scope of practice was developed to include all aspects of ICM standard competencies, such as independent antenatal care (ANC), labor care, postnatal care, initial stabilization of emergencies (including administration of medicines), contraceptive provision, cervical cancer screening and treatment, clinical management of rape, and first line management of common STIs. Administration of medicine became a particular challenge despite being an essential part of midwives' scope. The initial language used authorized midwives to administer medicines for midwifery care according to national guidelines. However, after deployment, many midwives were limited by managers who believed that non-doctors must have a specific medicine list. This type of resistance to midwives leading care provision was not limited to medicine administration but was experienced across multiple evidence-based midwifery interventions (e.g., avoidance of routine episiotomy, skin-to-skin care between mother and newborn, and stabilization of cases of eclampsia and postpartum hemorrhage [PPH] before referral).^18^ Thus, it was decided to revise the scope of practice to ensure midwives' use of lifesaving medicines.^24^ Three years after developing the medicine list for midwives, at the time of this writing, it was still awaiting final signatures. Until approved, midwives' ability to use medicines remains a gray area and limits their potential.

### Education

The goal of establishing quality midwifery education is to prepare competent midwives. Evidence-based instruction in the classroom, effective use of simulation, and quality clinical practice experience, ideally in accredited education programs, are the foundation. Continuing professional development and higher education maintain midwives' expertise and facilitate professional advancement after graduation.^3^

#### Diploma Program

**FIGURE 2:**
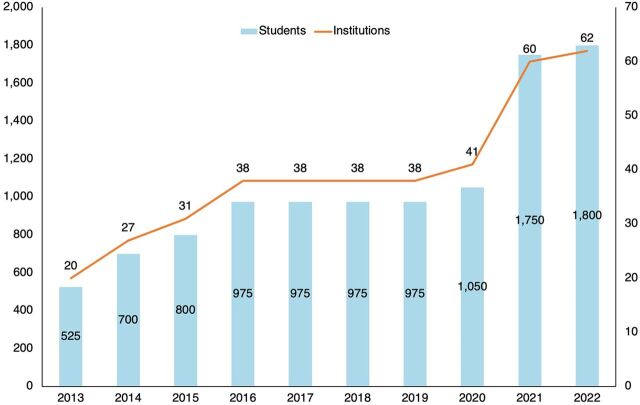
Growth in Midwifery Education Institutions and Students in Midwifery Diploma Program in Bangladesh, 2013–2022

A 3-year, ICM-aligned diploma in midwifery curriculum and syllabus were designed with the support of international academic experts from Auckland University for use in public and private sectors. In 2013, 525 students began the diploma program in 20 public nursing education institutes and 7 private sector institutes nationwide. Students complete 12 years of schooling and an entrance exam for admission. By 2022, the government and private sector had initiated 62 public and 105 private programs that are graduating 1,800 students annually (Figure 2).^10^ The number of midwives educated is based on the 20,000 midwives needed to meet WHO estimates of 1 midwife per 175 annual births,^25^ as well as existing deployment plans within the DGNM.^20^ As the number of midwives educated continues to increase—ensuring education quality—in the classroom, in labs, and at clinical practice sites^26^ is ever more important. UNFPA and academic partners are working with the government regulatory bodies to strengthen systems to improve the quality of education by training and mentoring faculty on both clinical topics and teaching pedagogy and providing faculty with opportunities to pursue further education in sexual and reproductive health and rights. For students, the diploma program has integrated the objective structured clinical assessment tool into diploma program skills labs^27^ and introduced mentorship at clinical practice sites to enable midwifery students to have the exposure and guidance for attaining necessary competencies.^19^

By 2022, the government and private sector had initiated 167 public and private programs that are graduating 1,800 midwifery students annually.

Education institutions receive authorization to initiate classes through a BNMC-led process. Although it is based on ICM curriculum standards, the checklist used to determine authorization does not align with global accreditation standards.^28^ In 2019, the curriculum was revised to include all aspects of comprehensive SRMNAH and improve the quality of education with a focus on critical thinking and simulation. Revisions were introduced through orientation sessions and faculty mentoring. Through project support, all public programs have received a package of midwifery instructional materials.

#### Upskilling Nursing Faculty to Teach Midwifery

DGNM provided a 1-month midwifery curriculum orientation to nursing faculty before launching the midwifery programs. However, gaps in faculty expertise remained after the orientations. To address these gaps, DGNM assigned faculty dedicated roles to either nursing or midwifery. In addition, DGNM provided ongoing training and mentoring with support from UNFPA through Dalarna and Aukland Universities and Save the Children International.^29^

In 2016, with UNFPA support, Dalarna University began delivering an international blended learning sexual and reproductive health (SRH) master's program for faculty allocated to midwifery to deepen faculty knowledge of SRMNAH, as well as strengthen teaching pedagogy and academic leadership. The program was delivered through a combination of web-based lectures, videos, in-person instruction, and online coaching. English language comprehension among midwifery faculty presented ongoing challenges that prompted offering translation and academic support. By December 2022, 150 midwifery faculty from 62 public education programs had completed the SRH master's degree program.^23^

To address identified gaps in mentoring, UNFPA through Dalarna and Aukland Universities and Save the Children International organized and provided faculty mentorship.^29^ Twenty designated faculty became peer mentors through an international online certificate course. Although continued support is needed until expert midwives can move into faculty roles, mentorship has contributed to improvements, including greater familiarity with comprehensive SRMNAH and increased faculty competence with using an Ambu bag and mask.^30^

#### Monitoring Dashboard

To support consistency across sites and share outcomes of ongoing monitoring and mentoring, a checklist was developed and served as a tool for mentors when documenting quality practices during visits to monitor quality improvement. The checklist was used to inform the development of a dashboard of education institution performance (Supplement). Dashboard elements include information on student facilities, management procedures, and teaching pedagogy, as well as quality of care and student oversight at clinical practice sites. The dashboard was initially thought to be shared with BNMC as a way to increase their monitoring, but this was not adopted. Currently, the dashboard is being used to guide peer mentors during visits and for program managers to oversee gaps.

#### Clinical Practice Experience

When the diploma program was launched in 2013, tertiary hospitals (without midwives) served as clinical practice sites. However, poor quality of care and competition for clinical experience between cadres (i.e., doctors, nurses, and midwives) were early challenges.^20^ These were considered a major barrier to quality education and are recognized as particular challenges in low- and middle-income countries.^27^^,^^31^ Two initiatives were undertaken to improve midwifery students' clinical practice experiences. First, rural hospitals with midwives were designated as clinical practice sites to enable modeling of midwifery-led care. Second, mentors were deployed through Save the Children International to all clinical practice sites to facilitate quality care.^32^ Mentors were medical doctors with specialized training in midwifery. Their roles straddled clinical care quality oversight and education of doctors, nurses, and hospital managers in evidence-based care in alignment with WHO standards and the midwife scope of practice.^33^^,^^34^ Their purpose was to advance evidence-based midwifery care at clinical sites and ensure students' exposure to all areas of quality, comprehensive SRMNAH. Mentors held education sessions with managers, providers, and students; organized weekly meetings with all staff to monitor progress; and delivered feedback and practical sessions to review skills and guidelines.^26^ Students tracked their clinical skill experiences in a personal logbook, specifying the number of cases attended of the 18 required evidence-based practices and an additional 25 services suggested for quality clinical experience. For example, attendance of 40 normal deliveries, 20 complicated labors, 100 newborn assessments, 100 postnatal checks of mothers, and 5 experiences managing obstetric emergencies were required. One hundred family planning counseling sessions, 5 intrauterine device insertions, and up to 10 experiences managing postpartum hemorrhage and eclampsia were suggested. Nursing supervisors reviewed and signed the logbooks. As more midwives are deployed and gain experience, they are beginning to hold supervisory positions over labor wards, allowing them to oversee midwifery students' practice experience.

However, despite significant success in enabling midwife-led quality care and adequate student supervision at clinical sites, some students still graduated without having proven their clinical competence.^35^ Care quality gaps at practice sites mirror those within the larger health system and are systemic. Students will replicate what they observe and practice.^20^ This experience suggests a need for greater acknowledgment in the global midwifery literature of care quality challenges at clinical education sites.

#### Higher Education

National midwifery Bachelor of Science and Master of Science programs were developed to create pathways for professional growth. Nurses—both educators and policymakers—have completed master's programs, and a select few have participated in international midwifery doctoral programs. The Bachelor of Science program in midwifery started in July 2022 with 76 students enrolled in 4 education institutions. The Master of Science program will be initiated in 2024 and will be led by doctoral candidates once they have completed their program.^23^

#### Continuing Education

Continuing education linked to licensing is planned and moving through approval processes. A detailed continuing professional development plan has been drafted and approved by BNMC and is awaiting approval from the MOHFW. The plan details the specific requirements for relicensing every 5 years. Government- and project-led in-person training are ongoing, and virtual training is available through both BMS and NGOs.^36^

#### Accreditation

In 2017, BNMC initiated an accreditation process. An initial quality assessment found gaps in the process, but follow-up was not formalized.^35^ After that, development partners from both nursing and midwifery worked together to finalize a national accreditation process. It was applied at 1 college where an external review identified significant needs for improvement. However, formal feedback given to the college was overwhelmingly positive. This gap in having the accreditation process respond to real issues is likely due to the hierarchical culture and corresponding need to maintain a positive public image, highlighting the importance of having a fully autonomous accreditation body.

### Association

A national professional association for midwifery is a core element of the profession. The association serves as a platform for midwives' engagement in health sector policy dialogue and helps raise midwifery's profile within countries. In addition, it enables midwives to connect with one another, have a unified voice, and access professional development opportunities.^3^^,^^4^

#### Formulation

Formed in 2010 through the support of UNFPA, BMS advocates for midwife participation in key policy and decision-making fora. First comprising nursing faculty, BMS was formed before the diploma program's initiation. This helped sensitize key players and kept Bangladesh recognized internationally as a country leading the introduction of professional midwives. However, having nurses instead of midwives hold leadership positions led to gaps in passion and vision in the early years. It also instigated a difficult handover once ICM-standard diploma midwives were available to move into leadership. After years of receiving capacity-building and organizational development assistance from UNFPA and beginning in 2016 from the UK Royal College of Midwives, BMS is now realizing its vision of becoming an active support to the midwifery profession. A twinning project between BMS and the Royal College of Midwives in the United Kingdom started in 2016 and strengthened BMS' organizational and leadership capacity.^10^

#### Elections

The transfer of power from nurse-midwives to diploma midwives in a 2018 election drew attention to powerful hierarchies in the nursing profession and biases against midwives' leadership competencies due to their youth and inexperience. The twinning project with the Royal College of Midwives facilitated the 2018 election, and the new diploma midwife leaders brought a palpable energy and commitment to the association. After the election, BMS raised awareness, prepared young leaders, engaged in policy dialogue, provided professional development, and gave midwives and students a voice. Sustainability and organizational effectiveness are still evolving. However, there is a strong sense of ownership, belonging, and vision among midwife members.^10^

### Workforce

Ensuring an adequate, competent, accessible midwifery workforce that can practice in a safe environment is essential to upholding women's rights. Countries must do the needed planning to meet SRMNAH needs, including for the poorest and most remote. In Bangladesh, the current number of government-deployed midwives is drastically lower than the 20,000 needed for a midwife for every woman based on conservative WHO estimates.^8^

#### Public Sector Deployment

The initial national deployment was planned for 3,000 midwives. Four midwives were to be assigned to each government subdistrict hospital (300,000–500,000 population) and 1 midwife to selected rural community facilities (30,000–50,000 population). In 2018, the first government deployment put 1,149 midwives in 342 of the country's 430 subdistrict hospitals.^12^ In 2021, a second deployment raised this number to 2,556 in 667 subdistrict hospitals. In 2022, 5,000 more midwife posts were created and are being filled in batches through 2026. This will allow midwives to be deployed to all facility levels, including tertiary-level medical colleges and district hospitals. An additional 20,000 more posts are needed and are under consideration by the Ministry of Health.

In 2018, the first government deployment put 1,149 midwives in 342 subdistrict hospitals, which, in 2021, increased to 2,556 midwives in 667 subdistrict hospitals.

#### Data Systems

Data on the impact of ICM-standard midwives are needed globally. National and project data collection systems that address SRMNAH care are crucial for gap identification and continuous improvement. In Bangladesh, several data collection templates have been designed and adapted over time for midwifery projects, allowing for important knowledge to be gathered. Where feasible, projects collect observation data on quality measures. These include proportion of births in which the following was observed: ANC services separated from general care, hydration and companion present during labor, correct use of partograph, immediate skin-to-skin provided for 1 hour, breastfeeding initiated within 1 hour, non-supine labor and delivery, delayed cord clamping, oxytocin and magnesium sulfate available in labor and delivery rooms, Ambu bag available in delivery room, postpartum family planning services available, and cases of postpartum hemorrhage and preeclampsia appropriately managed.

In addition, advocacy by UNFPA enabled indicators on midwifery to be integrated into the national health information system (DHIS2), thus allowing midwifery data to become part of national reports (Table 1).

**TABLE 1.** Indicators for Government-Deployed ICM Standard Midwife Service Delivery Data Gathered by National DHIS2

Indicators	Definition/Operational Definition
ANC by midwives	
ANC 1	1st visit: at 4 months/16 weeks.
ANC 2	2nd visit: at 6–7 months/24–28 weeks.
ANC 3	3rd visit: at 8 months/32 weeks.
ANC 4	4th visit: at 9 months/36 weeks.
Maternity care by midwives	
Births by midwives	Normal delivery is a physiologic process during which the fetus, membranes, umbilical cord, and placenta are expelled from the uterus.
Adolescent delivery (aged 15–19 years)	Clients who have normal delivery at the age of 15 to 19 years.
PPH coming from community	PPH cases coming from the community and managed and not referred.
PPH coming from community initial management and refer	Number of PPH cases coming from the community referred after initial stabilization.
Eclampsia managed locally at facilities with cesarean delivery capacity	Eclampsia cases coming from the community managed without referral at facilities with cesarean capacity.
Eclampsia initial management and refer	Number of eclampsia cases coming from the community and provided with the initial management and referred.
Newborn resuscitation	Neonatal resuscitation with Ambu bag.
PNC by midwives	Care of mother and newborn from 2 hours after delivery, up to 6 weeks.
Contraceptives by midwives	
IUCD	The IUCD is a small flexible device inserted into the uterine cavity to prevent pregnancy.
Injection	Contraceptive injection is a temporary family planning method for women in Bangladesh.
Oral pill/other	Oral contraceptive pills prevent pregnancy.
First trimester abortion by midwives	
Abortion is either medical or surgical and may be performed without a pregnancy test if menstruation is delayed.	
PAC by midwives	Services that are provided after spontaneous or induced abortion.
VIA screening	
Positive	Number of VIA tests performed by midwives with number of positive cases.
Negative	Number of VIA tests performed by midwives with number of negative cases.
GBV	
Violence against women is defined as any act of GBV that results in or is likely to result in physical, sexual, or psychological harm or suffering to women, including threats of such acts, coercion, or arbitrary deprivations of liberty whether occurring in public or in private life.	
Cases	Number of GBV cases seen by the midwives.

Abbreviations: ANC, antenatal care; GBV, gender-based violence; ICM, International Confederation of Midwives; IUCD, intrauterine contraceptive device; PAC, postabortion care; PNC, postnatal care; PPH, postpartum hemorrhage; VIA, visual inspection of the cervix with ascetic acid.

#### Private Sector

Registered midwives awaiting public sector deployment often take positions as nurses or paramedics in private hospitals. Designated midwife positions in private facilities are not common, but some exist. The private sector is less regulated than the public sector and is not required to report to the national health information system, making it much more difficult to track care quality. However, the most recent Bangladesh Health Facility Survey is indicative of lower quality SRH care in the private sector than in the public sector.^37^ Results show less readiness to provide comprehensive family planning and ANC in private hospitals and relatively equivalent readiness to provide normal delivery services with improvement needed across both hospital types (Table 2).

**TABLE 2.** Service Readiness of Different Hospital Types^a^

Service Area	Private Hospitals, %	Public Hospitals, %
FP		
Guidelines on FP	5	91
Staff trained on FP	28	88
Blood pressure apparatus available	95	94
Combined progestin-only oral pill	33	100
Progestin-only oral pill	11	94
Male condom	32	99
All 6 FP services	0	72
ANC		
Guidelines on ANC	7	61
Staff trained on ANC	26	87
Blood pressure apparatus	98	99
Hemoglobin testing capacity	82	79
Urine protein testing capacity	79	68
Iron or folic acid tablets	78	97
All 6 ANC services	2	32
Normal delivery		
Guidelines on emergency obstetric care	5	22
Staff trained in delivery care	16	57
Examination light	91	80
Delivery pack	94	87
Suction apparatus	89	57
Neonatal bag and mask	88	89
Partograph	22	42
Gloves	69	72
Injectable uterotonic oxytocin	72	61
Injectable antibiotic	70	59
Magnesium sulfate	45	28
Skin disinfectant	71	68
Intravenous fluids with infusion set	65	65
All 13 delivery services	0	0

Abbreviations: ANC, antenatal care; FP, family planning.

a Where midwives are deployed.

#### Humanitarian Response

Access to quality SRMNAH services reduces preventable maternal and newborn deaths in humanitarian and fragile settings where mortality is highest.^15^^,^^16^ Bangladesh has some of the most frequent and severe humanitarian crises globally, including natural disasters, refugees, and pandemics. Since 2016, diploma midwives have been a critical humanitarian cadre.^10^ Midwives were initially sent into cyclone response settings through NGO projects where they expanded service access and use. Their services were recognized, contributing to them feeling pride in their work and setting the stage for other humanitarian response deployments with NGOs.^38^

The concentration of health care services and the humanitarian response infrastructure in emergency program contexts (e.g., training on clinical management of rape for all SRMNAH providers) gave midwives significant professional experience that they later brought to their government posts.^39^ This was particularly notable during the large Rohingya refugee crisis. Given the emphasis on comprehensive sexual and reproductive health and rights within NGO-led programs and the training opportunities available, midwives deployed through NGO projects attained more relevant experience than those who were first deployed to private sector hospitals where they may not have been assigned to a midwifery role. The close contact between managers and midwives in humanitarian settings also allowed for feedback to the national education programs about gaps in new graduates' competencies and potential areas of strengthening for future education.

#### COVID-19 Pandemic

After the identification of the first COVID-19 case in Bangladesh, maternity service use dramatically declined in part due to staff absence and feeling overwhelmed.^40^^,^^41^ More than 200 project surge midwives were deployed to medical college, district, and subdistrict hospitals. A midwife-led telemedicine intervention was introduced in 5 districts.^42^ In many cases, midwives and mentors led the introduction of COVID-19 safety protocols for hospital triage systems and for establishing separate rooms for COVID-19 symptomatic mothers to receive ANC and deliver.^19^ Before and after surveys of 91 hospitals with midwives showed significant improvements across all measures of COVID-19 readiness.^42^

### Enabling Environment

An enabling environment is the context midwives need to practice effectively and achieve optimal outcomes.^3^^,^^4^ Originally, it was thought that strong regulation was adequate to enable the effective roll-out of midwifery-led care. However, it became clear over time that new midwives need on-site post-deployment support to succeed in improving SRMNAH quality and availability.^14^^,^^43^^,^^44^

In Bangladesh, gaps in vision and midwives' subordinate status in hospital hierarchies posed challenges. Although midwives were expected to make unprecedented changes to existing care systems, nurses, doctors, and managers were resistant to change due to their own limited awareness of quality SRMNAH and lack of understanding of differences between nurses' and midwives' roles.^45^^,^^46^ At the time of midwives' deployment, outdated and harmful practices (e.g., supine birth, routine episiotomy, and manual exploration of the uterus) were commonly performed. Evidence-based practices (e.g., delayed umbilical cord clamping, skin-to-skin contact, and Ambu bag use) were not routinely followed, and hesitance to manage obstetric emergencies at rural facilities was common.^47^ Once deployed, midwives were often placed in generalist nursing roles, while nurses tended to remain posted on maternity wards. Even when placed in maternity care roles, midwives' provision of emergency obstetric care, particularly for those coming from the community, was often discouraged by supervisors. Nurses felt midwives were too young to lead care provision. In addition, managers indicated that nurses may feel a sense of competition with the new midwives in part due to the potential loss of tips provided by patients for delivery care. As midwives did not accept tips from patients (their ICM-aligned pre-service education includes content on the right to health care for the poor), women and their families showed appreciation for both the free care and its quality.^17^ Midwives integrated most easily into hospital teams, led maternity care provision, and gained acceptance from communities where mentors were placed to provide support.

#### Facility Mentorship

Facility mentoring was an effective strategy for improving enabling environments. Mentors, who were young female doctors trained to champion midwives within their workplaces, visited each facility twice per month. With UNFPA support, Save the Children International and other implementing NGOs used an evidence-based low-dose, high-frequency mentoring approach.^27^ Facility mentors played a crucial role in building acceptance of the midwives and evidence-based practices among nurses, doctors, and hospital managers. Doctors were chosen as mentors because their professional status drew respect from the hospital managers, doctors, and nurses that the new midwives did not typically receive. This allowed the mentors to form peer relationships with managers and advocate for the value of the midwives.^48^ In a study from Bangladesh that compared hospitals with and without midwives and then compared these hospitals to those with and without mentors, midwives improved quality of care, but those who had mentors performed better.^16^

Facility mentoring was an effective strategy for improving enabling environments.

Over time, mentorship was shifted to the government. Initially funded by UNFPA, a new distinction of district SRH officers began conducting facility mentoring. Existing district public health nurses also added midwifery oversight to their duties and began tracking progress in care quality and midwives' ability to practice autonomously (Table 3).

**TABLE 3.** Sample Dashboard Used by Government District Public Health Nurses to Supervise Midwives at District Hospitals^a^

Hospital	Do the midwives provide autonomous ANC?	Are normal pregnancy cases only seen by a midwife for ANC?	Is an ANC card used for every mother?	Do the midwives distribute supplements (i.e., iron, folic acid, calcium)?	Do the midwives provide services in consultation with doctors (if needed)?	Do the midwives provide services during emergencies for initial stabilization or doctor referral?	Do the midwives manage labors independently?	Do at least 75% of women labor and deliver in upright and lateral positions?	Do the midwives provide continuous companionship/labor support?	Do the midwives ensure hydration and nutrition during labor for all women?	Is the Labor Care Guide used during every delivery?
Sharishabari	Yes	Yes	No	Yes	Yes	Yes	Yes	Yes	Yes	Yes	Yes
Bakshiganj	Yes	Yes	Yes	Yes	Yes	Yes	No	Yes	Yes	Yes	Yes
Madarganj	No	No	No	Yes	Yes	Yes	Yes	No	No	No	No
Islampur	Yes	Yes	Yes	Yes	Yes	Yes	No	Yes	Yes	Yes	Yes
Malandah	No	No	Yes	No	Yes	No	No	No	No	No	Yes
Deowanganj	Yes	Yes	Yes	Yes	Yes	Yes	Yes	Yes	Yes	Yes	Yes
Rajnagar	Yes	Yes	Yes	No	Yes	Yes	Yes	Yes	Yes	Yes	Yes
Sreemangal	Yes	Yes	Yes	No	Yes	Yes	Yes	Yes	Yes	Yes	No
Kamalganj	Yes	Yes	Yes	Yes	Yes	Yes	Yes	Yes	No	Yes	Yes
Kulaura	Yes	Yes	Yes	Yes	Yes	Yes	Yes	Yes	Yes	No	Yes

Abbreviation: ANC, antenatal care.

a This is a sample. No indicates follow-up needed.

Of the 64 facilities monitored by district public health nurses, the maternity wards in 62 facilities (97%) are run by midwives, births are managed independently by midwives in 63 facilities (98%), and at least 75% of women give birth upright or laterally in 57 facilities (89%).^49^

In humanitarian projects, midwives from different countries who are deployed to Bangladesh through NGO projects are used as mentors. Despite certain disadvantages (e.g., language), international mentors were a high-impact investment and aided in enabling midwives to implement the reproductive health minimum initial service package.^50^ As midwives in humanitarian settings matured, some moved into mentoring roles themselves. In some cases, national doctors were engaged to support the midwives to implement their full competencies.

#### Social Media

Widely available smartphones in Bangladesh have made social media ubiquitous. Social media was used iteratively over time to help midwives solve problems on the job and support the establishment of enabling environments by connecting midwives to one another and to national midwifery leadership. Facebook groups became a platform to connect deployed midwives, enabling midwives to regularly share experiences and UNFPA and government midwifery specialists to disseminate information on quality care. More than 5,000 midwives participated in these Facebook groups, posting thousands of photos and commentary on quality midwifery care practices, including managing postpartum hemorrhage and eclampsia.

## OUTCOMES AND IMPACT

### National and Subnational Deployment

**FIGURE 3:**
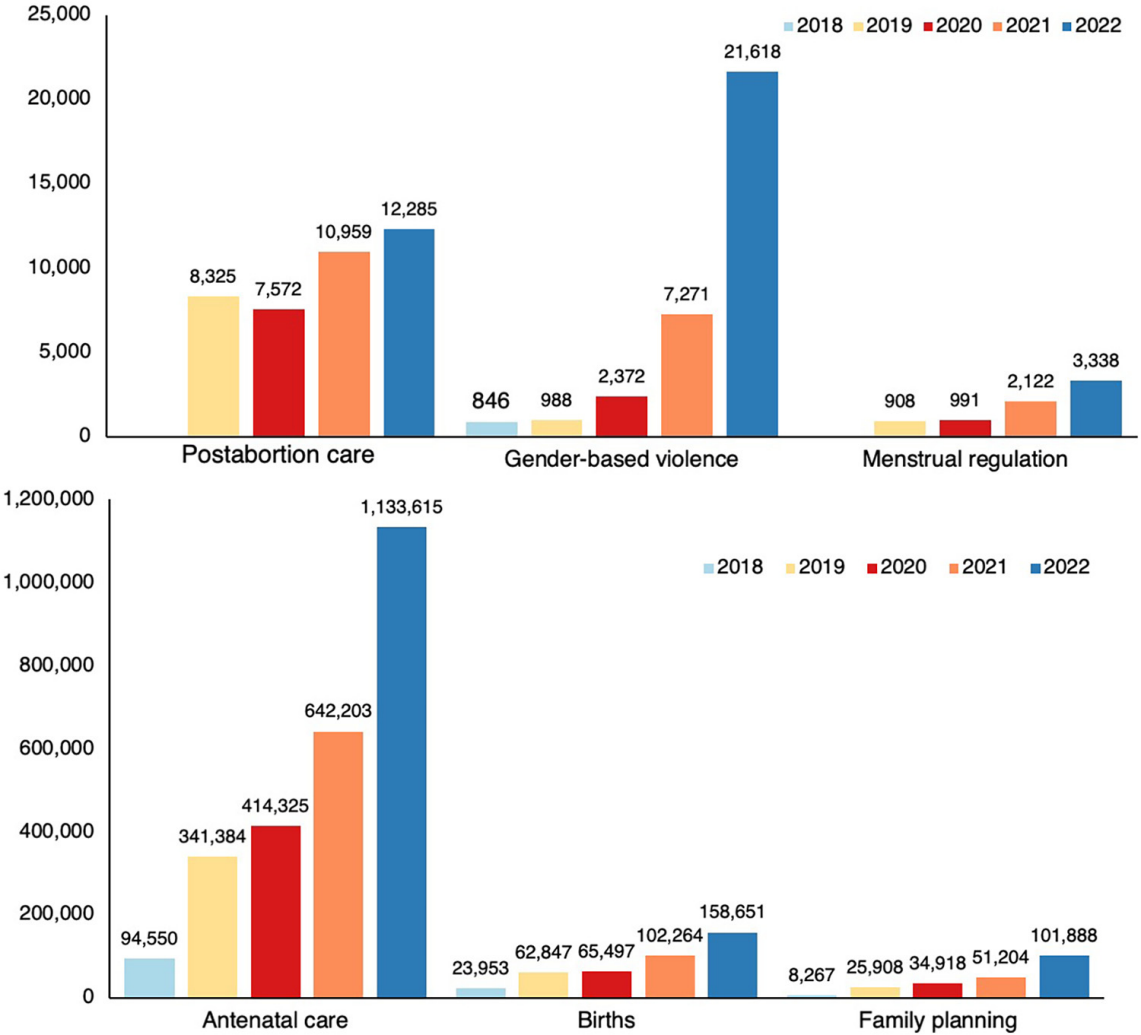
Sexual, Reproductive, Maternal, Neonatal, and Adolescent Health Services by Midwives in Bangladesh, 2018–2022 Source: United Nations Population Fund program data.

**FIGURE 4:**
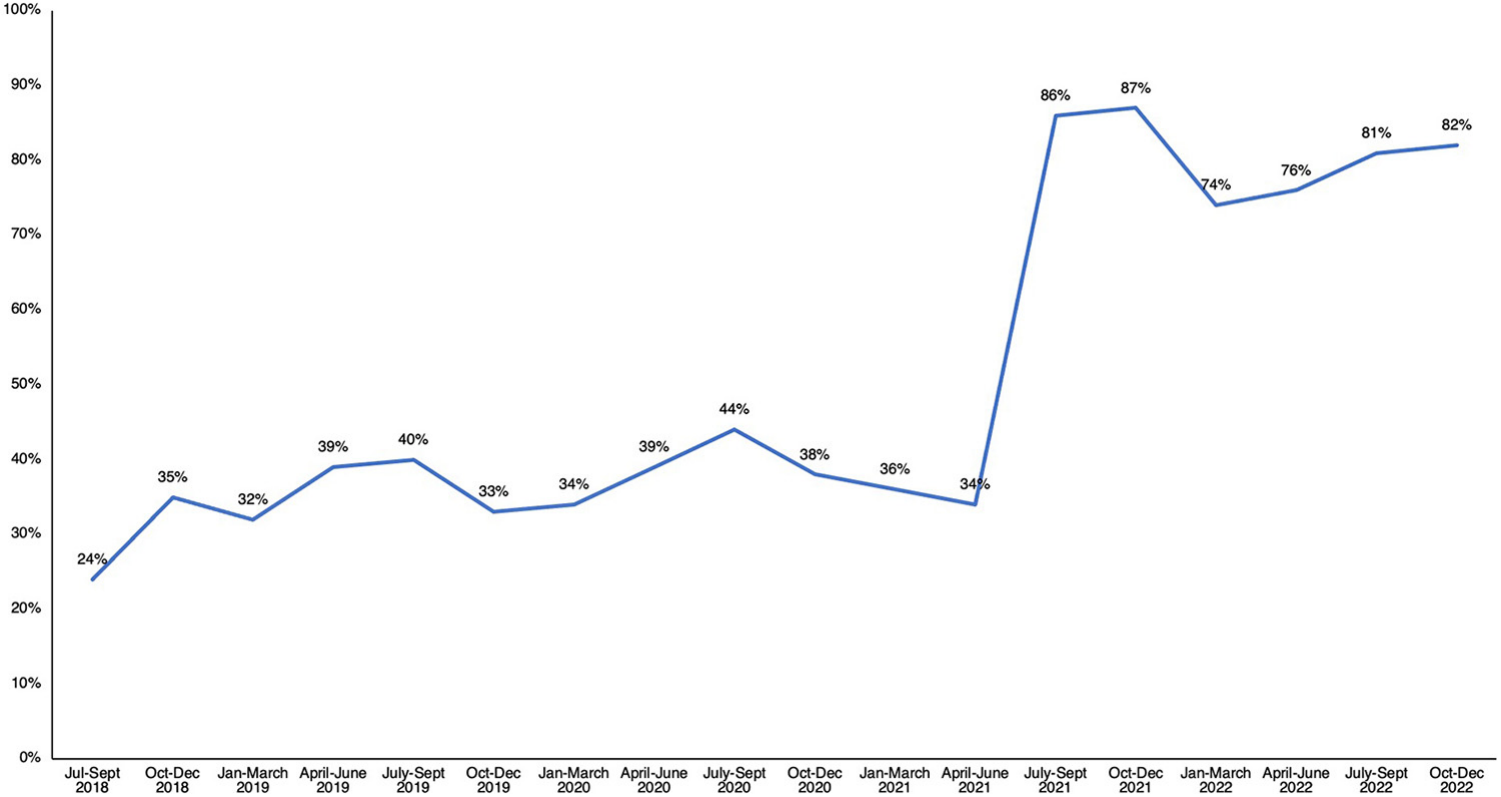
Percentage of Total Births Attended by Midwives in 378 Subdistrict Hospitals in Bangladesh Source: United Nations Population Fund program data.

**FIGURE 5:**
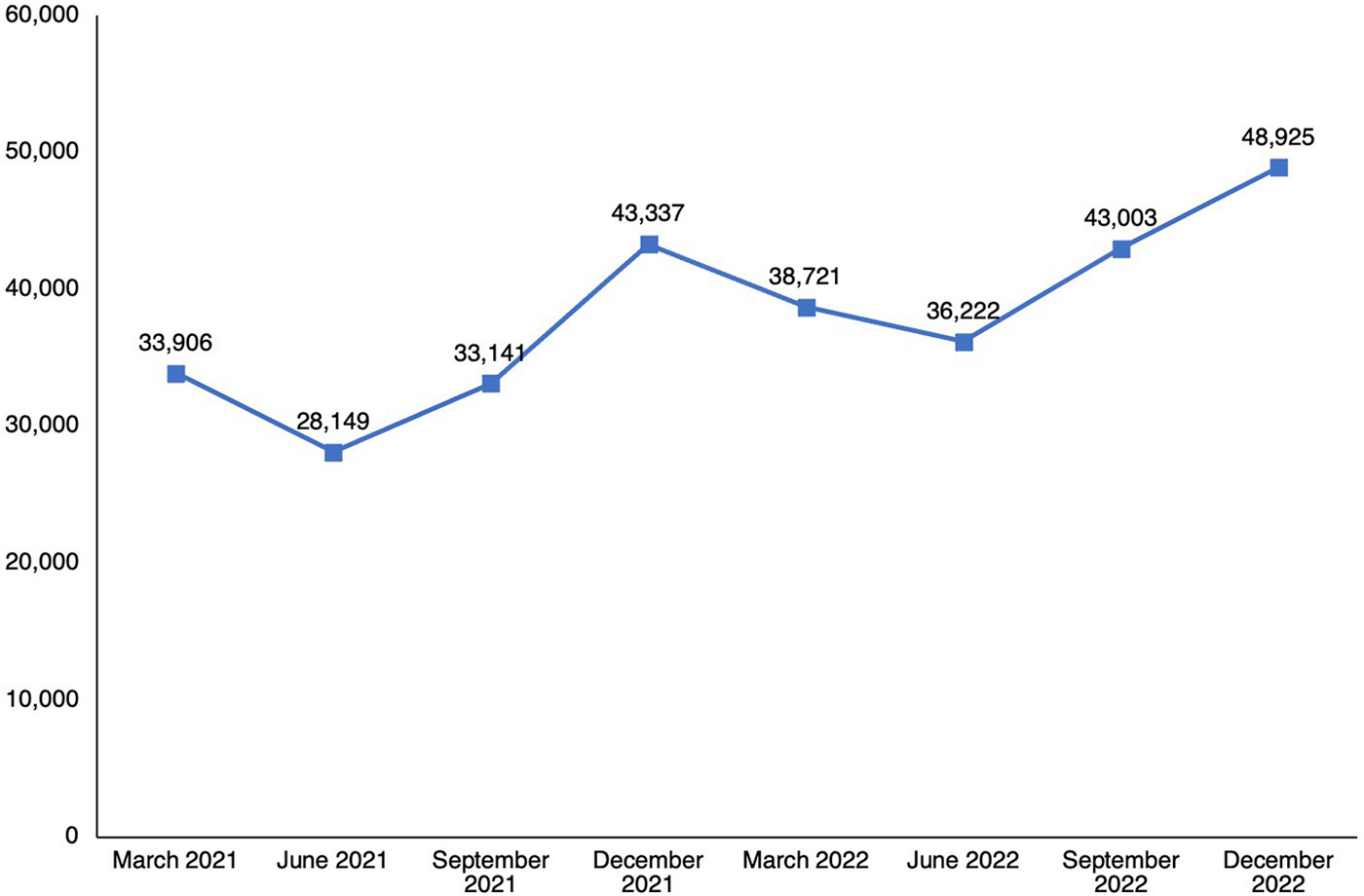
Total Births in 294 Subdistrict Hospitals With at Least 4 Midwives, Bangladesh Source: National health information system.

**FIGURE 6:**
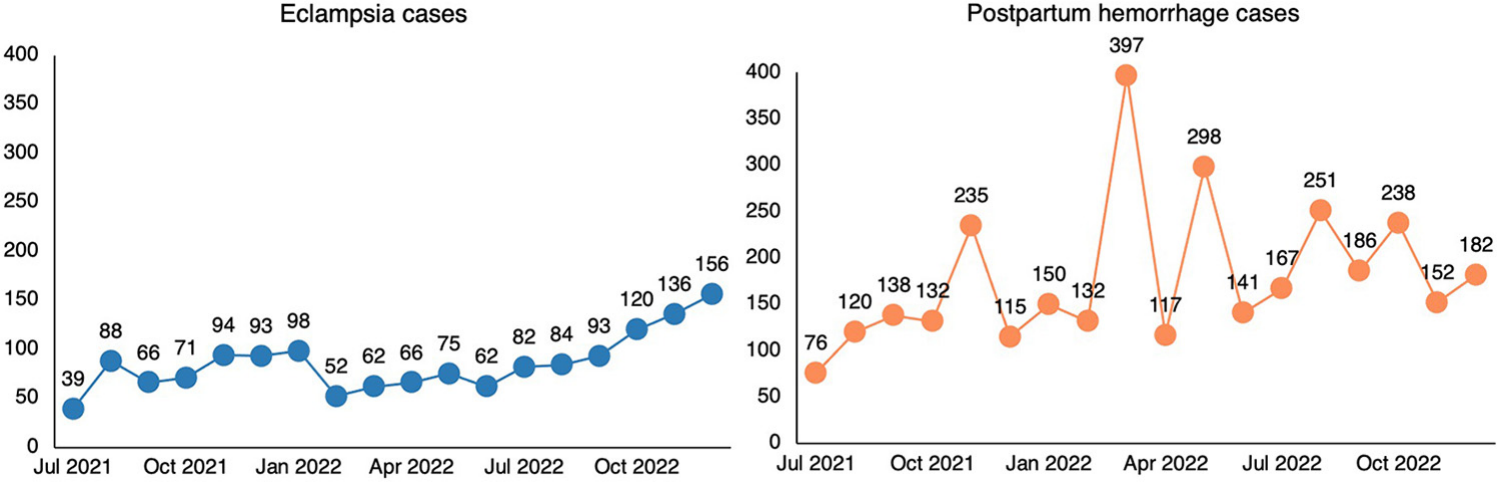
Eclampsia and Postpartum Hemorrhage Cases Treated by Midwives, July 2021 to December 2022, Bangladesh

Since 2018, government-deployed midwives have performed 2,626,077 ANC visits and attended 413,212 births (Figure 3). A 2022 survey of 153 subdistrict hospitals with midwives in 19 districts found that 90% of labor wards were run by midwives. Nationally, at facilities where midwives are deployed, they are attending around 80% of births (Figure 4), and the number of births they are attending is steadily increasing (Figure 5). Although midwives have started to implement all aspects of comprehensive SRH services, SRH service numbers are lower than maternity care service numbers (Figure 3). This results from non-maternity SRH services being considered less of a priority to make available and for midwives to provide these services. Data from the national health information system indicated that midwives managed a steadily increasing number of cases of PPH (from 39 to 156) and cases of eclampsia (from 76 to 182) between July 2021—when national reporting on these was initiated—and December 2022 (Figure 6). Four subnational studies have also been carried out using service data from selected facilities.

Nationally, at facilities where midwives are deployed, they are attending around 80% of births.

### Facility Comparison Groups

One study compared government subdistrict hospitals in Bangladesh without midwives to those with recently introduced midwives, both with and without facility mentoring. It examined whether the introduction of midwives (with and without mentorship) was associated with improved quality and availability of maternity care. The sample comprised 169 women who gave birth at 19 busy subdistrict hospitals and 473 women attending ANC. Quantitative analysis found that compared to hospitals without midwives, hospitals with midwives were significantly more likely to use an upright position for labor, delayed cord clamping, and skin-to-skin contact after birth, and hospitals with both midwives and mentoring were more likely to use these 3 practices, as well as ANC cards and partographs (Table 4).^18^

**TABLE 4.** Comparison of Results Between Government Subdistrict Hospitals in Bangladesh: Without Midwives, With Midwives, and With Midwives and Mentors

Measure	Without Midwives	With Midwives	Midwives and Mentors
Number of facilities ready for obstetric emergencies	3 of 7	1 of 6	4 of 6
Percentage of staff who valued, felt capable of using, and used evidence-based practices	68%	81%	92%
Number of evidence-based care practices used more than 50% of the time	4 of 8	6 of 8	8 of 8
Number of evidence-based practices with significantly greater use than in facilities without midwives (fixed effect logistic regression)	N/A (reference group)	3 of 8	5 of 8
Midwives' competence	Staff imagined that having midwives would improve service provision.	Among nurses and managers, some affirmed midwives' contribution, while some felt they were too inexperienced to be autonomous. Midwives expressed having the capacity to do more but not being allowed (e.g., some were not allowed to deliver babies).	Managers affirmed midwives' capacities and contribution and midwives stated they were proud to be midwives.
Separate antenatal care corners	Nonexistent	Transitioning to staffing antenatal care corners with midwives.	Antenatal care corners consistently staffed by midwives.
Management of obstetric emergencies	Nurses reported that they do not manage obstetric emergencies (commonly, patients were referred).	Midwives expressed confidence in managing obstetric emergencies but having limited autonomy so were not able to if another staff person decided to refer.	Midwives said they managed obstetric emergencies.

### Urban Hospital Study

**FIGURE 7:**
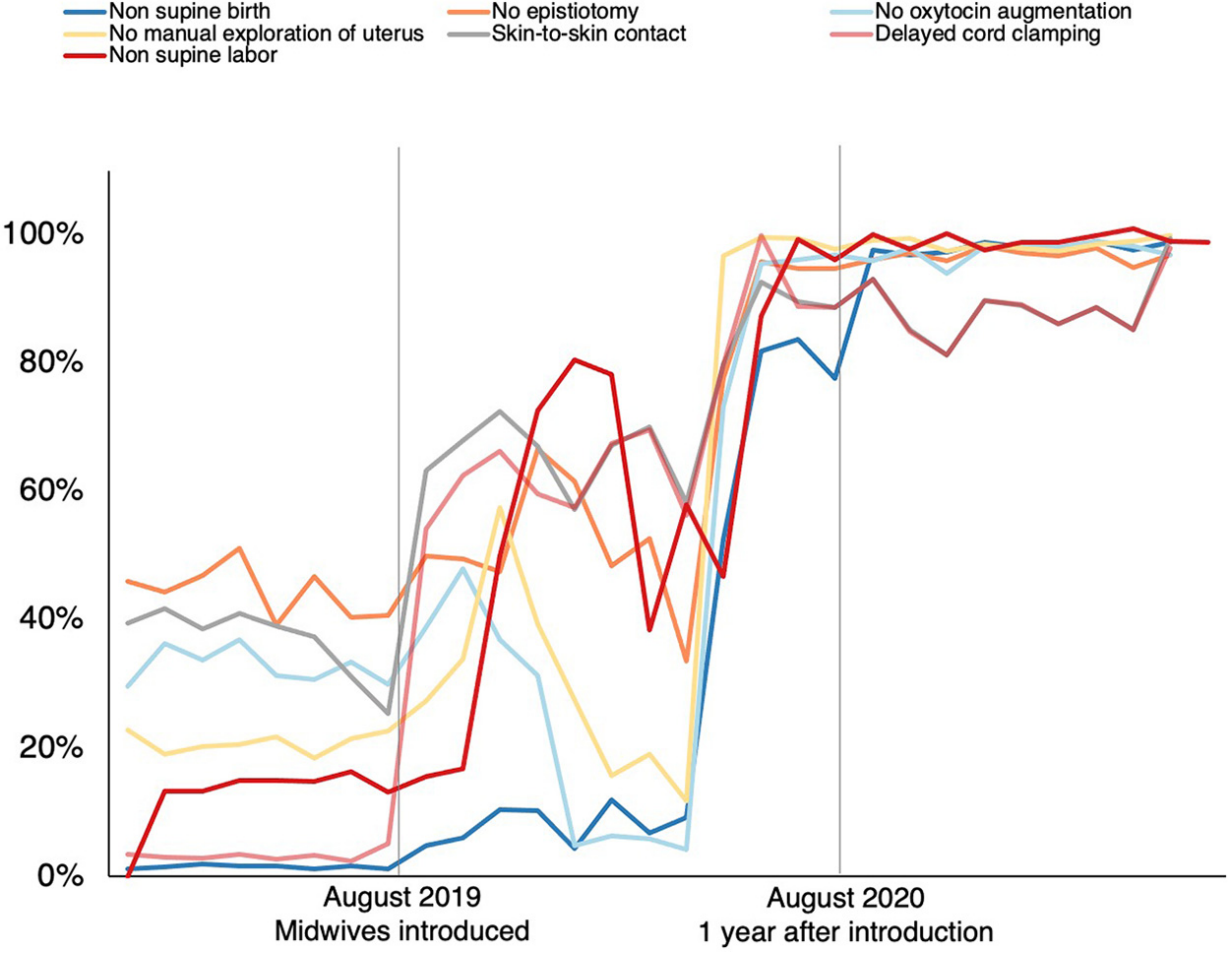
Midwives' Use of Evidence-Based Practices Before and After Introduction of Midwifery Service in 2 Teaching Hospitals in Dhaka, Bangladesh

Another study examined midwives' use of evidence-based practices at 2 high-capacity tertiary care teaching hospitals in Dhaka that serve more than 1,000 pregnant women each month. In August 2019, each hospital introduced a midwifery service, with 6–8 midwives and 1 facility mentor. An overall increase in evidence-based care was found immediately after the midwives' introduction. After 1 year, when midwives were attending upward of 85% of births, their use of all 7 evidence-based practices was significantly greater than in the period before the midwives' introduction (Figure 7).^19^

### Hospital Subset Analyses

Save the Children International analyzed facility readiness data from 47 subdistrict hospitals where newly graduated diploma midwives were first deployed by the government. Facility readiness increased across all measures between the 2018 baseline and the 2019 midline (Table 5).^29^

**TABLE 5.** Subdistrict Hospital Facility Readiness, Bangladesh

Indicators	Baseline, 2018 (N=47)	Midline, 2019 (N=47)
	No. (%)	No. (%)
Separate antenatal care and postnatal care corner available	27 (57)	45 (96)
Availability of oxytocin	19 (40)	38 (81)
Availability of magnesium sulfate	6 (13)	38 (81)
Visual inspective of the cervix with acetic acid service available	12 (26)	35 (74)
Postpartum family planning service available at inpatient and outpatient	15 (32)	45 (96)
Postabortion care service available	12 (26)	41 (87)
Separate duty roster for midwives	1 (2)	41 (87)

Save the Children International also analyzed observation data of care quality at clinical practice sites connected to midwifery education institutes between 2017 and 2019. Major improvements were seen in all 6 areas observed (Table 6).

**TABLE 6.** Care Quality at Clinical Practice Sites Connected to Educational Institutes, 2017–2019

Major Evidence-Based Practices	2017, % (N=87)	2018, % (N=657)	2019, % (N=652)
Correctness of partograph	7	51	62
Continuous companionship	67	87	94
Maintaining hydration and nutrition during labor	77	86	92
Non-supine, non-lithotomy birthing positioning	15	49	74
Delayed cord	61	76	87
Skin-to-skin contact for at least 1 hour	10	56	76

### Telemedicine Intervention

As part of the COVID-19 pandemic response, a telemedicine service for midwives was introduced in 2021 in 13 of 36 (36%) subdistrict hospitals in 6 districts supported under a donor project. Gender-based violence (GBV) screening and referral were integrated into the service. After 1 year of implementation, ANC service use in 31 facilities and facility births in all 36 had increased to levels before the pandemic.^42^ Also, in all 36 facilities, GBV sessions and referrals steadily increased from the start of the program, and PPH and eclampsia identification more than doubled from before the pandemic to after. Statistical testing showed significant increases in GBV service provision, ANC visits, PPH management, and postnatal care utilization and no significant increase in facility births or eclampsia management.^42^

## LESSONS LEARNED AND BEST PRACTICES

The experience of rolling out a new midwifery profession in Bangladesh shows that it is possible for a low-income country to introduce globally standard midwifery—that is distinct from nursing—to improve quality SRMNAH services in both humanitarian and development settings. Introducing midwives requires deployment strategies that meet workforce and enabling environment needs for both education and service. Implementation barriers and resistance to midwifery care were common and effectively addressed through ongoing on-site support. A full 20-year period is anticipated from the writing of the first strategic directions to midwifery education and leadership being spearheaded by midwives. However, this experience has shown that the first midwives can enter the workforce and start to improve quality in about half of that time if the groundwork is well laid and implementation support is given.

This experience has shown that the first midwives can enter the workforce and start to improve quality in about half of that time if the groundwork is well laid and implementation support is given.

The following are notable areas of recommended focus for other initiatives to develop a globally standard midwifery profession in a low- and middle-income country setting.

- **Separation of midwifery from nursing**: Ensure policy, regulation, and administration allow for a separate midwife profession. Separate midwifery education (including faculty), standard operating procedures, and posts are needed to reap the full benefits of the profession and to make real change in SRMNAH.
- **Midwives' use of medicines**: Vigilance to ensure midwives can administer medicines independently is needed. This is critical to saving lives and can meet significant resistance from doctors. For midwives to prevent maternal deaths, they must be able to administer medicine. In Bangladesh's case, program managers assumed that midwives would be able to use medicines. The resistance encountered is still being resolved. Addressing this should be a priority from the beginning of any efforts to establish a midwifery profession.
- **Teaching tools and pedagogy**: Ensure a separate midwifery curriculum with lesson plans and course content and be vigilant with monitoring their implementation. Capacity building on teaching pedagogy with emphasis on critical thinking is commonly needed to counter traditional rote memorization approaches. A strong curriculum is a priority, but syllabi, lesson plans, and predesigned instructional materials help in guiding faculty.
- **Simulation labs**: Simulation labs are essential for clinical education and may need extra attention if they are not yet implemented. New simulation staff may be needed, as well as organizational systems to support the faculty. Faculty may need orientation, training, and on-site mentoring.
- **Quality clinical practice experience**: Ensure that clinical education sites demonstrate quality, respectful comprehensive SRH care. Where there are gaps, prioritize strengthening them through mentoring. Students will learn what they observe and practice. Ensure that midwife students have experiences in all areas of comprehensive SRH, including evidence-based routine care, obstetric and newborn emergencies, health response to GBV, postabortion care, cervical cancer screening and treatment, sexually transmitted infections, and provision of contraceptives.
- **Continuing education linked to licensing**: Continuing education linked to licensing should be addressed early. This was not prioritized until fairly late in Bangladesh and still has not yet been realized. A system to connect the midwifery association and the regulatory body is needed for this and requires support.
- **On-site support and mentorship**: The WHO calls for on-site support to improve quality. In Bangladesh, both faculty and deployed midwives needed in-person mentorship to make the significant changes needed. Ensuring there are champions for quality SRMNAH may be critical for change to be accepted.
- **Midwifery and comprehensive SRMNAH**: Build comprehensive SRMNAH into education and midwife-led services early. Non-maternity SRMNAH services may be new and need extra attention to establish. Confusion about midwives' non-maternity SRMNAH expertise can contribute to their being seen as exclusively focused on maternity care.
- **Social media**: Social media and virtual groups can be effective means of sensitizing midwives, managers, and faculty on care quality. Social media enable sharing of successes and challenges due to their large reach and ease of sharing images. In Bangladesh, more than 5,000 midwives and all faculty have joined virtual groups.
- **Association leadership**: Ensure the midwifery association is led by ICM-standard midwives as soon as this is possible. In Bangladesh, the passion for midwifery was stronger among midwives than among nurses.

## CONCLUSION

A profession of globally standard midwives deployed into the national health care system will improve the quality and availability of SRMNAH services. Bangladesh, as well as several other countries, has succeeded in developing a midwifery profession that is distinct from nursing, and the poorest women are benefiting from the results. All contexts will have specific considerations, but many countries can benefit from Bangladesh's guidance.

## Supplementary Material

GHSP-D-23-00081-supplement.pdf

## Notes

**Peer Reviewed**

**First published online:** October 18, 2023.
