# Supplementary material for: Health System Strengthening Through Professional Midwives in Bangladesh: Best Practices, Challenges, and Successes
Source: Glob Health Sci Pract. 2023 Oct 30;11(5):e2300081. doi: 10.9745/GHSP-D-23-00081 (PMC10615233; doi:10.9745/GHSP-D-23-00081)

**Supplement to:** Begum F, Ara R, Islam A, Marriott S, Williams A, Anderson R. Health system strengthening through professional midwives in Bangladesh: best practices, challenges, and successes. *Glob Health Sci Pract*. 2023;11(5):e2300081. https://doi.org/10.9745/GHSP-D-23-00081

## SUPPLEMENT. Faculty Mentoring Checklist

| **REQUIRED INFORMATION** | | |
|---|---|---|
| Name of data collector | | |
| Organization of data collector | | |
| Name of nursing college/institute | | |
| Date of the site visit | | |
| **Infrastructure** | **Seen** | **Comments** |
| Adequate number of student classrooms and equipped skills labs | | |
| Adequate student accommodations | | |
| Skills lab open daily 8am-8pm (students confirm) | | |
| Computer lab open daily 8am-8pm (students confirm) | | |
| Students report WIFI access | | |
| **Teaching** | **Seen** | **Comments** |
| Classes follow the curriculum (taught subject observed) 100% | | |
| Faculty introduces themselves and the topic of the lesson, outlines the lesson objectives | | |
| Faculty uses interactive teaching methods and teaching aids, asking questions and provide feedback and checks that students understand the lesson | | |
| Faculty conducts all Objective Structured Clinical Examination (OSCE) items correctly (observed) as per curriculum | | |
| _Faculty provide scenario or instruction before conducting OSCE | | |
| _Faculty included basic steps, such as-Greetings/praise the mother, hand washing | | |
| _Faculty demonstrate clearly for student to observe | | |
| _Faculty helps student understand evidence-based practice | | |
| _Faculty identified the student's gap and find out what the students know and proceed from there | | |
| _Faculty provided constructive feedback in an appropriate manner | | |
| Guest lecturer following curriculum and syllabus (observed or confirmed guest lecturer lesson plan or students notes) | | |
| Skill lab practice (confirmed by attendance register book and students) | | |
| Library study (confirmed by attendance register book and students) | | |
| Computer lab practice (confirmed by attendance register book and students) | | |
| Faculty arrange monthly staff meeting | | |
| **Clinical** | **Seen** | **Comments** |
| Institute maintains clinical coordination | | |
| Coordination meetings held quarterly or as needed with clinical site for curriculum implementation | | |
| Senior midwifery faculty record documentation | | |
| Meeting agenda | | |
| Attendance list | | |
| Minutes | | |
| Decisions and actions | | |
| Progress of decision action (previous meeting) | | |
| Faculty at hospital clinical site whenever students are there (students confirm and duty roster of clinical teaching) | | |
| Faculty coordinate students' experiences with clinical site (nursing supervisor/ward in-charge confirms) | | |
| Students report they have adequate opportunity to attend births | | |
| Students report they have adequate opportunity to provide EmONC (i.e., PPH/PEE) care. | | |
| Students report they have adequate opportunity to get SRHR experiences (i.e., GBV, CMR, FP) | | |
| Students report quality supervision when in clinical (students confirm) | | |
| Faculty sign students' log book (observe in log book) | | |
| Harmful behavior observed when in clinical site | | |

**Supplement to:** Begum F, Ara R, Islam A, Marriott S, Williams A, Anderson R. Health system strengthening through professional midwives in Bangladesh: best practices, challenges, and successes. *Glob Health Sci Pract*. 2023;11(5):e2300081. https://doi.org/10.9745/GHSP-D-23-00081

| | | |
|---|---|---|
| Nursing supervisor/facility director appears supportive of midwifery education (observed administrative support and barriers) | | |
| Midwifery students placement in attached hospitals/UHC/MLC site | | |

## Select Elements From the Faculty Mentor Dashboard

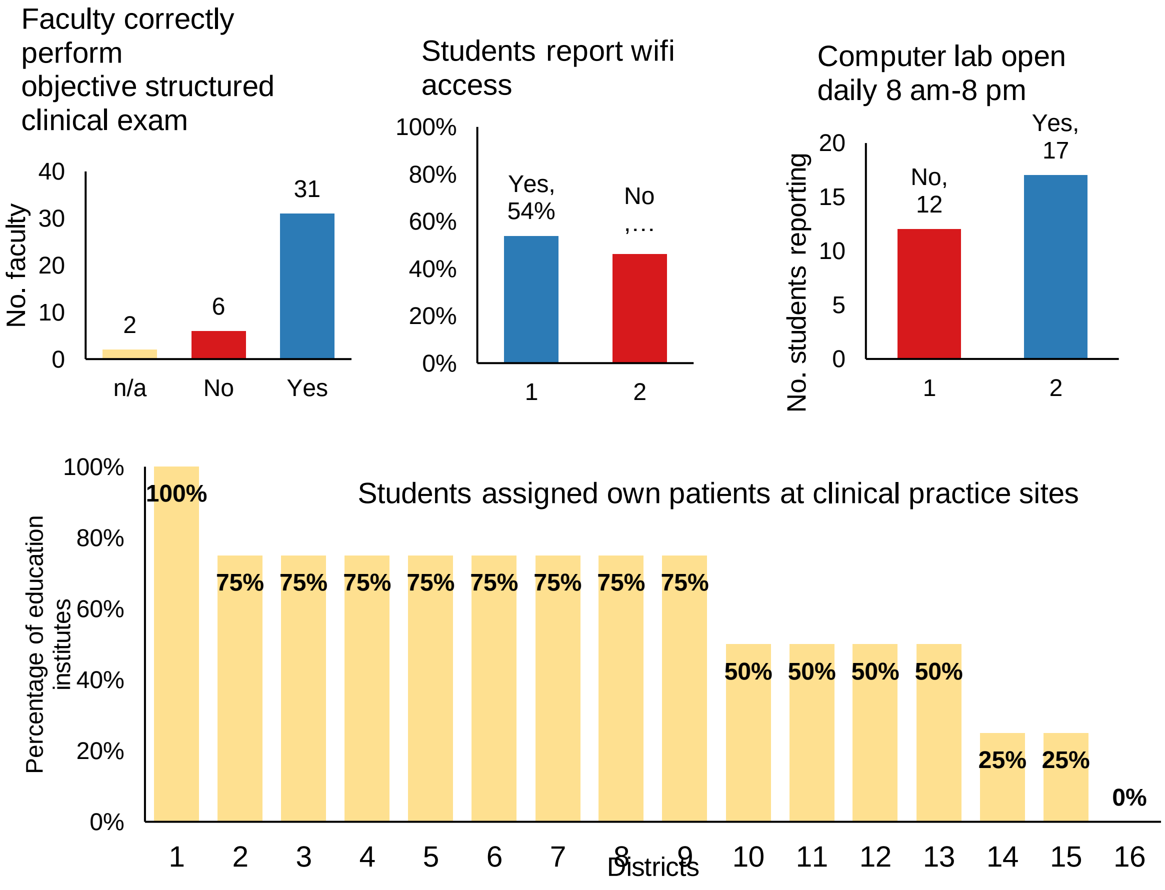

Supplement: GHSP-D-23-00081-supplement.pdf [file GHSP-D-23-00081-supplement.pdf]
